# Bis(2-amino-6-methyl-1,3-benzothia­zole-κ*N* ^3^)bis­(4-nitro­benzoato-κ*O* ^1^)zinc

**DOI:** 10.1107/S1600536811022331

**Published:** 2011-06-18

**Authors:** Xue-Tong Sun, Xiu-Guang Wang, Xiao-Jun Zhao

**Affiliations:** a College of Chemistry, Tianjin Key Laboratory of Structure and Performance for Functional Molecules, Tianjin Normal University, Tianjin 300387, People’s Republic of China

## Abstract

In the title mononuclear complex, [Zn(C_7_H_4_NO_4_)_2_(C_8_H_8_N_2_S)_2_], the Zn^II^ atom is coordinated by two N atoms from two 2-amino-6-methyl-1,3-benzothia­zole and by two carboxylate O atoms from two 4-nitro­benzoate ligands, adopting a slightly distorted tetra­hedral coordination geometry. In the crystal, inter­molecular N—H⋯O hydrogen bonds between the amino group of 2-amino-6-methyl-1,3-benzothia­zole and the carboxyl­ate group of 4-nitro­benzoate link these discrete mononuclear units into a one-dimensional supra­molecular chain extending parallel to [100].

## Related literature

For the properties of metal complexes with amino­benzothia­zole and its derivatives, see: Sun & Cui (2008 ▶); Chen *et al.* (2008 ▶); Kovalska *et al.* (2006 ▶); Batista *et al.* (2007 ▶); Marconato *et al.* (1998 ▶).
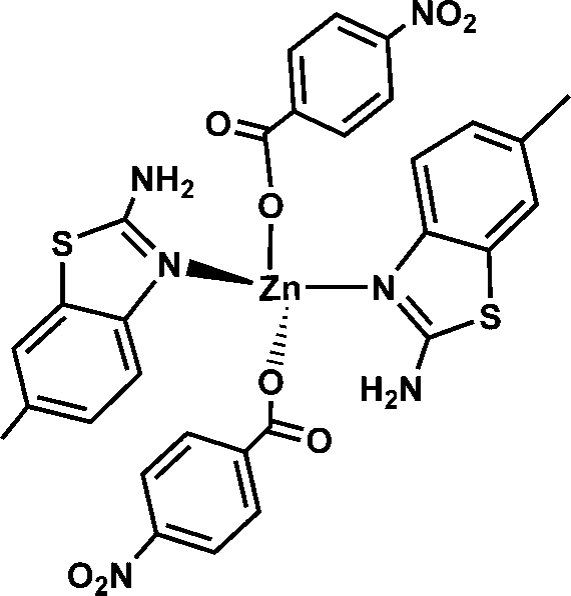


## Experimental

### 

#### Crystal data

[Zn(C_7_H_4_NO_4_)_2_(C_8_H_8_N_2_S)_2_]
*M* *_r_* = 726.04
Monoclinic, 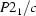

*a* = 13.2240 (8) Å
*b* = 10.7369 (7) Å
*c* = 21.8863 (13) Å
β = 96.099 (1)°
*V* = 3089.9 (3) Å^3^
*Z* = 4
Mo *K*α radiation
μ = 0.99 mm^−1^
*T* = 296 K
0.05 × 0.04 × 0.03 mm

#### Data collection

Bruker APEXII CCD diffractometer
Absorption correction: multi-scan (*SADABS*; Sheldrick, 1996 ▶) *T*_min_ = 0.952, *T*_max_ = 0.971
15119 measured reflections
5418 independent reflections
4724 reflections with *I* > 2σ(*I*)
*R*_int_ = 0.028

#### Refinement

*R*[*F* ^2^ > 2σ(*F* ^2^)] = 0.036
*wR*(*F* ^2^) = 0.101
*S* = 1.05
5418 reflections
426 parameters
H-atom parameters constrained
Δρ_max_ = 1.24 e Å^−3^
Δρ_min_ = −0.42 e Å^−3^

### 

Data collection: *APEX2* (Bruker, 2003 ▶); cell refinement: *SAINT* (Bruker, 2001 ▶); data reduction: *SAINT*; program(s) used to solve structure: *SHELXS97* (Sheldrick, 2008 ▶); program(s) used to refine structure: *SHELXL97* (Sheldrick, 2008 ▶); molecular graphics: *SHELXTL* (Sheldrick, 2008 ▶) and *DIAMOND* (Brandenburg & Berndt, 1999 ▶); software used to prepare material for publication: *SHELXL97*.

## Supplementary Material

Crystal structure: contains datablock(s) I, global. DOI: 10.1107/S1600536811022331/go2014sup1.cif

Structure factors: contains datablock(s) I. DOI: 10.1107/S1600536811022331/go2014Isup2.hkl

Additional supplementary materials:  crystallographic information; 3D view; checkCIF report

## Figures and Tables

**Table 1:** Selected bond lengths (Å)

Zn1—O6	1.9489 (18)
Zn1—O1	1.9717 (18)
Zn1—N3	2.036 (2)
Zn1—N1	2.054 (2)

**Table 2:** Hydrogen-bond geometry (Å, °)

*D*—H⋯*A*	*D*—H	H⋯*A*	*D*⋯*A*	*D*—H⋯*A*
N2—H2*A*⋯O1	0.86	2.27	3.008 (3)	144
N2—H2*B*⋯O5^i^	0.86	2.05	2.845 (3)	153
N4—H4*A*⋯O6	0.86	2.20	2.971 (3)	149
N4—H4*B*⋯O2^ii^	0.86	2.05	2.860 (3)	156

Symmetry codes: (i) 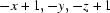; (ii) 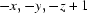.

**Fig. 1.**
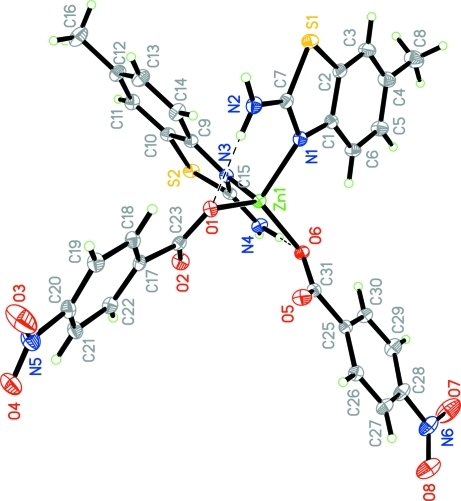
The molecular structure of (I). Displacement ellipsoids are drawn at the 30% probability level. Dashed lines indicate hydrogen bonds.

**Fig. 2.**
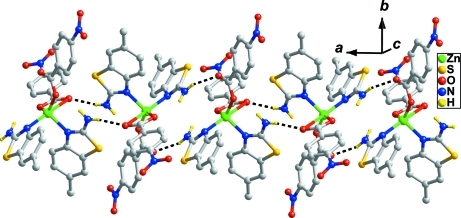
Part of the one-dimensional chain of (I) formed by N—H···O hydrogen bonds interactions. Only H atoms involving hydrogen bonds are included.

## supplementary crystallographic information

### Comment

Organic compounds containing benzothiazole and their metal complexes are of optical, biological, and pharmaceutical importance used extensively as electroluminescent device, fluorescent probes for DNA and corrosion inhibitors (Kovalska *et al.* 2006; Batista *et al.* 2007; Marconato *et al.* 1998). As our continuing investigation on the coordination behavior of the benzothiazole ligand (Chen *et al.* 2008), herein, we report the structure of a mononuclear complex, I.

The molecular structure of the title mononuclear complex is shown in Fig.1 and selected bond lengths and angles are listed in Table 1. The Zn^II^ atom in the mononuclear stucture of I exhibits a slightly distorted tetrahedral coordination geometry involving two thiazole N atoms from two different 2-amino-6-methyl-1,3-benzothiazole ligands and two monodentate carboxylate O atoms from two separate 4-nitrobenzolate anions. Both ligands act as typically terminal ligands to coordinate to Zn^II^ ion in monodentate mode with the intramolecular N—H···O hydrogen bonds between amino group of neutral 2-amino-6-methyl-1,3-benzothiazole and anionic 4-nitrobenzolate ligand stabilized the molecular structure.

In the crystal structure, intermolecular N—H···O hydrogen bonds between amino group of neutral 2-amino-6-methyl-1,3-benzothiazole and anionic 4-nitrobenzolate ligands link the discrete mononuclear entities into a one-dimensional supramolecular chain (Fig.2 and Table 2).

### Experimental

To an ethanol solution (6.0 ml) containing 2-amino-6-methyl-1,3-benzothiazole (32.8 mg, 0.2 mmol) and 4-nitrobenzoic acid (33.4 mg, 0.2 mmol) was dropwise added an aqueous solution (4.0 ml) of ZnCl_2_ (13.6 mg, 0.1 mmol) with constant stirring. The reaction was stirred at room temperature for about 20 min and the precipitate was filtered off, leaving the filtrate to evaporate at room temperature. Yellow block-shaped crystals were obtained with five days. Yield: 50% based on Zn^II^ salt. Anal. Calcd. for C_30_H_24_N_6_O_8_S_2_Zn: C, 49.63; H, 3.33; N, 11.57%. Found: C, 49.60; H, 3.31; N, 11.61%.

### Refinement

Refinement of F2 against ALL reflections. The weighted *R*-factor *wR* and goodness of fit S are based on F2, conventional *R*-factors *R* are based on F, with F set to zero for negative F2. The threshold expression of F2 > 2σ(F2) is used only for calculating *R*-factors(gt) *etc*. and is not relevant to the choice of reflections for refinement. *R*-factors based on F2 are statistically about twice as large as those based on F, and R– factors based on ALL data will be even larger.

### Crystal data

**Table:**

[Zn(C_7_H_4_NO_4_)_2_(C_8_H_8_N_2_S)_2_]	*F*(000) = 1488
*M**_r_* = 726.04	*D*_x_ = 1.561 Mg m^−^^3^
Monoclinic, *P*2_1_/*c*	Mo *K*α radiation, λ = 0.71073 Å
*a* = 13.2240 (8) Å	Cell parameters from 7342 reflections
*b* = 10.7369 (7) Å	θ = 2.7–27.7°
*c* = 21.8863 (13) Å	µ = 0.99 mm^−^^1^
β = 96.099 (1)°	*T* = 296 K
*V* = 3089.9 (3) Å^3^	Block, yellow
*Z* = 4	0.05 × 0.04 × 0.03 mm

### Data collection

**Table:**

Bruker APEXII CCD diffractometer	5418 independent reflections
Radiation source: fine-focus sealed tube	4724 reflections with *I* > 2σ(*I*)
graphite	*R*_int_ = 0.028
φ and ω scans	θ_max_ = 25.0°, θ_min_ = 1.6°
Absorption correction: multi-scan (*SADABS*; Sheldrick, 1996)	*h* = −15→11
*T*_min_ = 0.952, *T*_max_ = 0.971	*k* = −10→12
15119 measured reflections	*l* = −22→26

### Refinement

**Table:**

Refinement on *F*^2^	Primary atom site location: structure-invariant direct methods
Least-squares matrix: full	Secondary atom site location: difference Fourier map
*R*[*F*^2^ > 2σ(*F*^2^)] = 0.036	Hydrogen site location: inferred from neighbouring sites
*wR*(*F*^2^) = 0.101	H-atom parameters constrained
*S* = 1.05	*w* = 1/[σ^2^(*F*_o_^2^) + (0.0476*P*)^2^ + 4.110*P*] where *P* = (*F*_o_^2^ + 2*F*_c_^2^)/3
5418 reflections	(Δ/σ)_max_ = 0.001
426 parameters	Δρ_max_ = 1.24 e Å^−^^3^
0 restraints	Δρ_min_ = −0.42 e Å^−^^3^

### Special details

**Table:**

Geometry. All e.s.d.'s (except the e.s.d. in the dihedral angle between two l.s. planes) are estimated using the full covariance matrix. The cell e.s.d.'s are taken into account individually in the estimation of e.s.d.'s in distances, angles and torsion angles; correlations between e.s.d.'s in cell parameters are only used when they are defined by crystal symmetry. An approximate (isotropic) treatment of cell e.s.d.'s is used for estimating e.s.d.'s involving l.s. planes.
Refinement. Refinement of *F*^2^ against ALL reflections. The weighted *R*-factor *wR* and goodness of fit *S* are based on *F*^2^, conventional *R*-factors *R* are based on *F*, with *F* set to zero for negative *F*^2^. The threshold expression of *F*^2^ > σ(*F*^2^) is used only for calculating *R*-factors(gt) *etc*. and is not relevant to the choice of reflections for refinement. *R*-factors based on *F*^2^ are statistically about twice as large as those based on *F*, and *R*- factors based on ALL data will be even larger.A small void of about 37Å3 was ignored and was not dealt with SQUEEZE in *PLATON*, because it is so small that no solvent molecules can be kept in.

### Fractional atomic coordinates and isotropic or equivalent isotropic displacement parameters (Å^2^)

**Table:**

	*x*	*y*	*z*	*U*_iso_*/*U*_eq_	
Zn1	0.26944 (2)	0.00802 (3)	0.480986 (13)	0.02098 (11)	
S1	0.47182 (6)	0.30130 (7)	0.40799 (3)	0.03297 (19)	
S2	−0.01802 (5)	0.24653 (7)	0.48211 (3)	0.02783 (17)	
O1	0.34638 (13)	−0.01142 (17)	0.56266 (8)	0.0247 (4)	
O2	0.19228 (14)	−0.0698 (2)	0.58293 (9)	0.0302 (5)	
O3	0.5423 (2)	−0.2293 (3)	0.84770 (12)	0.0759 (10)	
O4	0.40207 (17)	−0.3229 (2)	0.85604 (10)	0.0459 (6)	
O5	0.30961 (15)	−0.27205 (19)	0.47934 (9)	0.0323 (5)	
O6	0.20619 (13)	−0.12869 (17)	0.43234 (8)	0.0244 (4)	
O7	−0.0259 (2)	−0.5840 (3)	0.24530 (15)	0.0772 (10)	
O8	0.0226 (2)	−0.7233 (3)	0.31379 (15)	0.0694 (8)	
N1	0.36676 (16)	0.1045 (2)	0.43113 (10)	0.0233 (5)	
N2	0.49494 (17)	0.1583 (2)	0.50904 (10)	0.0294 (5)	
H2A	0.4785	0.0988	0.5323	0.035*	
H2B	0.5445	0.2072	0.5214	0.035*	
N3	0.15791 (16)	0.1385 (2)	0.48208 (10)	0.0226 (5)	
N4	0.02296 (17)	0.0328 (2)	0.42585 (11)	0.0272 (5)	
H4A	0.0622	−0.0258	0.4156	0.033*	
H4B	−0.0410	0.0308	0.4135	0.033*	
N5	0.4538 (2)	−0.2505 (3)	0.83008 (12)	0.0405 (7)	
N6	0.0226 (2)	−0.6164 (3)	0.29378 (15)	0.0495 (8)	
C1	0.3274 (2)	0.1474 (3)	0.37255 (12)	0.0244 (6)	
C2	0.3728 (2)	0.2560 (3)	0.35280 (13)	0.0284 (6)	
C3	0.3393 (2)	0.3119 (3)	0.29694 (14)	0.0358 (7)	
H3	0.3691	0.3853	0.2852	0.043*	
C4	0.2610 (2)	0.2571 (3)	0.25899 (15)	0.0381 (7)	
C5	0.2186 (2)	0.1459 (3)	0.27823 (14)	0.0345 (7)	
H5	0.1673	0.1078	0.2525	0.041*	
C6	0.2504 (2)	0.0908 (3)	0.33415 (13)	0.0282 (6)	
H6	0.2208	0.0172	0.3458	0.034*	
C7	0.44390 (19)	0.1748 (3)	0.45406 (12)	0.0257 (6)	
C8	0.2202 (3)	0.3160 (4)	0.19792 (16)	0.0505 (9)	
H8A	0.2012	0.4008	0.2046	0.076*	
H8B	0.1617	0.2703	0.1804	0.076*	
H8C	0.2718	0.3139	0.1703	0.076*	
C9	0.1747 (2)	0.2472 (3)	0.51684 (12)	0.0243 (6)	
C10	0.0875 (2)	0.3181 (3)	0.52264 (13)	0.0266 (6)	
C11	0.0913 (2)	0.4282 (3)	0.55639 (13)	0.0308 (6)	
H11	0.0323	0.4733	0.5602	0.037*	
C12	0.1845 (2)	0.4698 (3)	0.58424 (14)	0.0339 (7)	
C13	0.2717 (2)	0.3993 (3)	0.57735 (14)	0.0359 (7)	
H13	0.3343	0.4279	0.5953	0.043*	
C14	0.2679 (2)	0.2894 (3)	0.54497 (13)	0.0312 (7)	
H14	0.3269	0.2437	0.5419	0.037*	
C15	0.06120 (19)	0.1267 (2)	0.46081 (12)	0.0228 (6)	
C16	0.1951 (3)	0.5880 (3)	0.62144 (16)	0.0459 (8)	
H16A	0.1288	0.6198	0.6269	0.069*	
H16B	0.2317	0.6487	0.6003	0.069*	
H16C	0.2314	0.5709	0.6609	0.069*	
C17	0.32872 (19)	−0.0954 (3)	0.66182 (12)	0.0228 (6)	
C18	0.4305 (2)	−0.0734 (3)	0.68280 (12)	0.0267 (6)	
H18	0.4710	−0.0274	0.6589	0.032*	
C19	0.4716 (2)	−0.1198 (3)	0.73917 (13)	0.0306 (6)	
H19	0.5390	−0.1041	0.7539	0.037*	
C20	0.4098 (2)	−0.1901 (3)	0.77294 (12)	0.0300 (6)	
C21	0.3074 (2)	−0.2099 (3)	0.75424 (13)	0.0305 (6)	
H21	0.2670	−0.2555	0.7784	0.037*	
C22	0.2671 (2)	−0.1600 (3)	0.69863 (13)	0.0277 (6)	
H22	0.1982	−0.1697	0.6858	0.033*	
C23	0.2843 (2)	−0.0555 (3)	0.59863 (12)	0.0236 (6)	
C25	0.18095 (19)	−0.3394 (3)	0.40249 (12)	0.0230 (6)	
C26	0.1996 (2)	−0.4654 (3)	0.41508 (13)	0.0271 (6)	
H26	0.2463	−0.4882	0.4479	0.032*	
C27	0.1490 (2)	−0.5567 (3)	0.37901 (14)	0.0336 (7)	
H27	0.1611	−0.6407	0.3873	0.040*	
C28	0.0805 (2)	−0.5203 (3)	0.33078 (15)	0.0364 (7)	
C29	0.0623 (2)	−0.3960 (3)	0.31583 (14)	0.0364 (7)	
H29	0.0177	−0.3741	0.2818	0.044*	
C30	0.1120 (2)	−0.3053 (3)	0.35279 (13)	0.0285 (6)	
H30	0.0993	−0.2215	0.3444	0.034*	
C31	0.23768 (19)	−0.2413 (3)	0.44193 (12)	0.0231 (6)	

### Atomic displacement parameters (Å^2^)

**Table:**

	*U*^11^	*U*^22^	*U*^33^	*U*^12^	*U*^13^	*U*^23^
Zn1	0.01631 (17)	0.02239 (18)	0.02357 (18)	0.00094 (12)	−0.00095 (12)	−0.00048 (12)
S1	0.0306 (4)	0.0337 (4)	0.0346 (4)	−0.0124 (3)	0.0033 (3)	−0.0041 (3)
S2	0.0185 (3)	0.0242 (4)	0.0404 (4)	0.0044 (3)	0.0012 (3)	0.0016 (3)
O1	0.0204 (9)	0.0301 (11)	0.0233 (9)	0.0020 (8)	0.0007 (8)	0.0001 (8)
O2	0.0186 (10)	0.0376 (12)	0.0328 (11)	0.0003 (8)	−0.0050 (8)	0.0015 (9)
O3	0.0495 (16)	0.119 (3)	0.0528 (16)	−0.0286 (17)	−0.0247 (13)	0.0367 (17)
O4	0.0456 (13)	0.0576 (16)	0.0341 (12)	−0.0051 (12)	0.0026 (10)	0.0146 (11)
O5	0.0272 (10)	0.0308 (11)	0.0360 (11)	−0.0024 (9)	−0.0101 (9)	0.0026 (9)
O6	0.0221 (9)	0.0225 (10)	0.0284 (10)	−0.0001 (8)	0.0010 (8)	−0.0011 (8)
O7	0.082 (2)	0.065 (2)	0.075 (2)	0.0077 (16)	−0.0390 (17)	−0.0271 (16)
O8	0.0672 (19)	0.0440 (17)	0.095 (2)	−0.0024 (14)	−0.0005 (16)	−0.0192 (16)
N1	0.0185 (11)	0.0266 (12)	0.0244 (11)	−0.0008 (9)	0.0000 (9)	−0.0019 (9)
N2	0.0215 (11)	0.0353 (14)	0.0303 (13)	−0.0038 (10)	−0.0016 (10)	−0.0061 (11)
N3	0.0181 (11)	0.0233 (12)	0.0261 (11)	0.0027 (9)	0.0010 (9)	0.0011 (9)
N4	0.0157 (11)	0.0276 (13)	0.0373 (13)	0.0010 (9)	−0.0026 (9)	−0.0018 (10)
N5	0.0344 (15)	0.0565 (18)	0.0290 (14)	−0.0043 (13)	−0.0041 (11)	0.0049 (13)
N6	0.0456 (17)	0.0400 (18)	0.061 (2)	0.0002 (14)	−0.0041 (15)	−0.0193 (15)
C1	0.0217 (13)	0.0252 (14)	0.0268 (14)	0.0011 (11)	0.0049 (11)	−0.0009 (11)
C2	0.0268 (14)	0.0284 (15)	0.0305 (15)	−0.0046 (12)	0.0059 (12)	−0.0063 (12)
C3	0.0393 (17)	0.0306 (17)	0.0376 (17)	−0.0060 (14)	0.0049 (14)	0.0050 (13)
C4	0.0400 (17)	0.0360 (18)	0.0377 (17)	−0.0017 (14)	0.0010 (14)	0.0054 (14)
C5	0.0320 (16)	0.0353 (17)	0.0344 (16)	−0.0044 (13)	−0.0049 (13)	0.0024 (13)
C6	0.0258 (14)	0.0262 (15)	0.0316 (15)	−0.0035 (12)	−0.0010 (12)	0.0025 (12)
C7	0.0188 (13)	0.0304 (15)	0.0285 (14)	−0.0008 (11)	0.0057 (11)	−0.0068 (12)
C8	0.058 (2)	0.051 (2)	0.0410 (19)	−0.0048 (18)	0.0004 (17)	0.0123 (17)
C9	0.0240 (14)	0.0223 (14)	0.0265 (14)	0.0023 (11)	0.0018 (11)	0.0017 (11)
C10	0.0242 (14)	0.0240 (14)	0.0312 (15)	0.0035 (11)	0.0009 (11)	0.0040 (12)
C11	0.0322 (15)	0.0265 (15)	0.0334 (15)	0.0103 (12)	0.0024 (12)	0.0006 (12)
C12	0.0378 (17)	0.0258 (15)	0.0367 (17)	0.0067 (13)	−0.0034 (13)	−0.0024 (13)
C13	0.0318 (16)	0.0333 (17)	0.0402 (17)	0.0016 (13)	−0.0069 (13)	−0.0046 (14)
C14	0.0246 (14)	0.0326 (16)	0.0348 (16)	0.0072 (12)	−0.0036 (12)	−0.0041 (13)
C15	0.0209 (13)	0.0213 (14)	0.0263 (14)	0.0025 (11)	0.0028 (11)	0.0049 (11)
C16	0.051 (2)	0.0349 (18)	0.048 (2)	0.0092 (16)	−0.0126 (16)	−0.0103 (15)
C17	0.0212 (13)	0.0246 (14)	0.0225 (13)	0.0039 (11)	0.0014 (10)	−0.0048 (11)
C18	0.0223 (13)	0.0327 (16)	0.0252 (14)	0.0001 (12)	0.0038 (11)	−0.0009 (12)
C19	0.0219 (14)	0.0403 (17)	0.0283 (15)	−0.0017 (12)	−0.0031 (11)	−0.0027 (13)
C20	0.0325 (15)	0.0352 (17)	0.0216 (14)	0.0010 (13)	0.0000 (12)	0.0001 (12)
C21	0.0282 (15)	0.0379 (17)	0.0261 (14)	−0.0029 (13)	0.0052 (12)	−0.0007 (13)
C22	0.0222 (13)	0.0322 (16)	0.0281 (14)	−0.0008 (12)	−0.0005 (11)	−0.0039 (12)
C23	0.0227 (14)	0.0214 (14)	0.0258 (14)	0.0037 (11)	−0.0007 (11)	−0.0031 (11)
C25	0.0190 (13)	0.0241 (14)	0.0260 (14)	0.0006 (11)	0.0029 (10)	−0.0008 (11)
C26	0.0234 (14)	0.0285 (15)	0.0288 (15)	0.0040 (12)	0.0006 (11)	0.0007 (12)
C27	0.0309 (16)	0.0258 (15)	0.0434 (17)	0.0043 (13)	0.0009 (13)	−0.0067 (13)
C28	0.0322 (16)	0.0332 (17)	0.0423 (18)	0.0004 (13)	−0.0027 (14)	−0.0130 (14)
C29	0.0338 (16)	0.0376 (18)	0.0349 (16)	0.0050 (14)	−0.0103 (13)	−0.0052 (14)
C30	0.0263 (14)	0.0263 (15)	0.0317 (15)	0.0020 (12)	−0.0024 (12)	−0.0011 (12)
C31	0.0195 (13)	0.0263 (15)	0.0235 (13)	−0.0012 (11)	0.0020 (11)	0.0010 (11)

### Geometric parameters (Å, °)

**Table:**

Zn1—O6	1.9489 (18)	C8—H8A	0.9600
Zn1—O1	1.9717 (18)	C8—H8B	0.9600
Zn1—N3	2.036 (2)	C8—H8C	0.9600
Zn1—N1	2.054 (2)	C9—C14	1.393 (4)
S1—C2	1.754 (3)	C9—C10	1.399 (4)
S1—C7	1.755 (3)	C10—C11	1.392 (4)
S2—C10	1.750 (3)	C11—C12	1.389 (4)
S2—C15	1.754 (3)	C11—H11	0.9300
O1—C23	1.287 (3)	C12—C13	1.401 (4)
O2—C23	1.238 (3)	C12—C16	1.507 (4)
O3—N5	1.214 (3)	C13—C14	1.375 (4)
O4—N5	1.216 (3)	C13—H13	0.9300
O5—C31	1.232 (3)	C14—H14	0.9300
O6—C31	1.289 (3)	C16—H16A	0.9600
O7—N6	1.230 (4)	C16—H16B	0.9600
O8—N6	1.229 (4)	C16—H16C	0.9600
N1—C7	1.324 (3)	C17—C22	1.390 (4)
N1—C1	1.409 (3)	C17—C18	1.396 (4)
N2—C7	1.327 (3)	C17—C23	1.506 (4)
N2—H2A	0.8600	C18—C19	1.387 (4)
N2—H2B	0.8600	C18—H18	0.9300
N3—C15	1.320 (3)	C19—C20	1.383 (4)
N3—C9	1.398 (3)	C19—H19	0.9300
N4—C15	1.332 (4)	C20—C21	1.388 (4)
N4—H4A	0.8600	C21—C22	1.384 (4)
N4—H4B	0.8600	C21—H21	0.9300
N5—C20	1.472 (4)	C22—H22	0.9300
N6—C28	1.474 (4)	C25—C30	1.392 (4)
C1—C6	1.389 (4)	C25—C26	1.397 (4)
C1—C2	1.401 (4)	C25—C31	1.510 (4)
C2—C3	1.391 (4)	C26—C27	1.386 (4)
C3—C4	1.388 (4)	C26—H26	0.9300
C3—H3	0.9300	C27—C28	1.373 (4)
C4—C5	1.403 (4)	C27—H27	0.9300
C4—C8	1.525 (4)	C28—C29	1.389 (4)
C5—C6	1.384 (4)	C29—C30	1.387 (4)
C5—H5	0.9300	C29—H29	0.9300
C6—H6	0.9300	C30—H30	0.9300
			
O6—Zn1—O1	124.45 (8)	C12—C11—H11	120.5
O6—Zn1—N3	104.78 (8)	C10—C11—H11	120.5
O1—Zn1—N3	111.52 (8)	C11—C12—C13	118.8 (3)
O6—Zn1—N1	110.27 (8)	C11—C12—C16	122.3 (3)
O1—Zn1—N1	104.06 (8)	C13—C12—C16	118.9 (3)
N3—Zn1—N1	98.91 (9)	C14—C13—C12	122.3 (3)
C2—S1—C7	89.64 (13)	C14—C13—H13	118.9
C10—S2—C15	89.45 (13)	C12—C13—H13	118.9
C23—O1—Zn1	106.99 (16)	C13—C14—C9	119.3 (3)
C31—O6—Zn1	120.34 (16)	C13—C14—H14	120.4
C7—N1—C1	110.8 (2)	C9—C14—H14	120.4
C7—N1—Zn1	125.95 (18)	N3—C15—N4	124.7 (2)
C1—N1—Zn1	117.15 (16)	N3—C15—S2	114.8 (2)
C7—N2—H2A	120.0	N4—C15—S2	120.4 (2)
C7—N2—H2B	120.0	C12—C16—H16A	109.5
H2A—N2—H2B	120.0	C12—C16—H16B	109.5
C15—N3—C9	111.4 (2)	H16A—C16—H16B	109.5
C15—N3—Zn1	127.29 (19)	C12—C16—H16C	109.5
C9—N3—Zn1	120.47 (17)	H16A—C16—H16C	109.5
C15—N4—H4A	120.0	H16B—C16—H16C	109.5
C15—N4—H4B	120.0	C22—C17—C18	119.9 (2)
H4A—N4—H4B	120.0	C22—C17—C23	118.6 (2)
O3—N5—O4	122.9 (3)	C18—C17—C23	121.5 (2)
O3—N5—C20	118.0 (3)	C19—C18—C17	120.3 (3)
O4—N5—C20	119.1 (2)	C19—C18—H18	119.8
O8—N6—O7	123.4 (3)	C17—C18—H18	119.8
O8—N6—C28	118.5 (3)	C20—C19—C18	118.2 (3)
O7—N6—C28	118.0 (3)	C20—C19—H19	120.9
C6—C1—C2	119.2 (3)	C18—C19—H19	120.9
C6—C1—N1	125.7 (2)	C19—C20—C21	122.6 (3)
C2—C1—N1	115.1 (2)	C19—C20—N5	119.4 (3)
C3—C2—C1	121.6 (3)	C21—C20—N5	117.9 (3)
C3—C2—S1	129.1 (2)	C22—C21—C20	118.2 (3)
C1—C2—S1	109.4 (2)	C22—C21—H21	120.9
C4—C3—C2	119.4 (3)	C20—C21—H21	120.9
C4—C3—H3	120.3	C21—C22—C17	120.5 (3)
C2—C3—H3	120.3	C21—C22—H22	119.7
C3—C4—C5	118.5 (3)	C17—C22—H22	119.7
C3—C4—C8	121.4 (3)	O2—C23—O1	123.2 (2)
C5—C4—C8	120.0 (3)	O2—C23—C17	119.7 (2)
C6—C5—C4	122.4 (3)	O1—C23—C17	117.0 (2)
C6—C5—H5	118.8	C30—C25—C26	119.7 (3)
C4—C5—H5	118.8	C30—C25—C31	120.5 (2)
C5—C6—C1	118.9 (3)	C26—C25—C31	119.8 (2)
C5—C6—H6	120.6	C27—C26—C25	120.6 (3)
C1—C6—H6	120.6	C27—C26—H26	119.7
N1—C7—N2	124.4 (3)	C25—C26—H26	119.7
N1—C7—S1	115.1 (2)	C28—C27—C26	118.4 (3)
N2—C7—S1	120.4 (2)	C28—C27—H27	120.8
C4—C8—H8A	109.5	C26—C27—H27	120.8
C4—C8—H8B	109.5	C27—C28—C29	122.6 (3)
H8A—C8—H8B	109.5	C27—C28—N6	119.0 (3)
C4—C8—H8C	109.5	C29—C28—N6	118.4 (3)
H8A—C8—H8C	109.5	C30—C29—C28	118.6 (3)
H8B—C8—H8C	109.5	C30—C29—H29	120.7
C14—C9—N3	126.5 (2)	C28—C29—H29	120.7
C14—C9—C10	118.8 (3)	C29—C30—C25	120.1 (3)
N3—C9—C10	114.7 (2)	C29—C30—H30	120.0
C11—C10—C9	121.8 (3)	C25—C30—H30	120.0
C11—C10—S2	128.6 (2)	O5—C31—O6	124.9 (2)
C9—C10—S2	109.6 (2)	O5—C31—C25	119.6 (2)
C12—C11—C10	119.1 (3)	O6—C31—C25	115.4 (2)
			
O6—Zn1—O1—C23	−61.77 (19)	S2—C10—C11—C12	−178.7 (2)
N3—Zn1—O1—C23	65.32 (18)	C10—C11—C12—C13	0.0 (5)
N1—Zn1—O1—C23	171.00 (17)	C10—C11—C12—C16	179.9 (3)
O1—Zn1—O6—C31	−22.1 (2)	C11—C12—C13—C14	−1.2 (5)
N3—Zn1—O6—C31	−151.97 (19)	C16—C12—C13—C14	178.9 (3)
N1—Zn1—O6—C31	102.47 (19)	C12—C13—C14—C9	1.3 (5)
O6—Zn1—N1—C7	−157.5 (2)	N3—C9—C14—C13	179.5 (3)
O1—Zn1—N1—C7	−21.9 (2)	C10—C9—C14—C13	−0.2 (4)
N3—Zn1—N1—C7	93.1 (2)	C9—N3—C15—N4	179.4 (2)
O6—Zn1—N1—C1	52.8 (2)	Zn1—N3—C15—N4	−11.3 (4)
O1—Zn1—N1—C1	−171.65 (18)	C9—N3—C15—S2	−0.7 (3)
N3—Zn1—N1—C1	−56.7 (2)	Zn1—N3—C15—S2	168.56 (13)
O6—Zn1—N3—C15	7.2 (2)	C10—S2—C15—N3	0.1 (2)
O1—Zn1—N3—C15	−129.9 (2)	C10—S2—C15—N4	−180.0 (2)
N1—Zn1—N3—C15	121.1 (2)	C22—C17—C18—C19	2.5 (4)
O6—Zn1—N3—C9	175.59 (19)	C23—C17—C18—C19	−174.8 (3)
O1—Zn1—N3—C9	38.5 (2)	C17—C18—C19—C20	1.4 (4)
N1—Zn1—N3—C9	−70.6 (2)	C18—C19—C20—C21	−3.7 (5)
C7—N1—C1—C6	175.9 (3)	C18—C19—C20—N5	174.2 (3)
Zn1—N1—C1—C6	−29.9 (3)	O3—N5—C20—C19	4.8 (5)
C7—N1—C1—C2	−3.3 (3)	O4—N5—C20—C19	−172.1 (3)
Zn1—N1—C1—C2	150.81 (19)	O3—N5—C20—C21	−177.2 (3)
C6—C1—C2—C3	3.2 (4)	O4—N5—C20—C21	5.9 (4)
N1—C1—C2—C3	−177.5 (3)	C19—C20—C21—C22	1.9 (5)
C6—C1—C2—S1	−176.4 (2)	N5—C20—C21—C22	−176.0 (3)
N1—C1—C2—S1	2.9 (3)	C20—C21—C22—C17	2.1 (4)
C7—S1—C2—C3	179.2 (3)	C18—C17—C22—C21	−4.3 (4)
C7—S1—C2—C1	−1.3 (2)	C23—C17—C22—C21	173.1 (3)
C1—C2—C3—C4	−1.8 (5)	Zn1—O1—C23—O2	−5.9 (3)
S1—C2—C3—C4	177.7 (2)	Zn1—O1—C23—C17	171.76 (18)
C2—C3—C4—C5	−0.5 (5)	C22—C17—C23—O2	7.0 (4)
C2—C3—C4—C8	178.8 (3)	C18—C17—C23—O2	−175.7 (3)
C3—C4—C5—C6	1.6 (5)	C22—C17—C23—O1	−170.7 (2)
C8—C4—C5—C6	−177.7 (3)	C18—C17—C23—O1	6.6 (4)
C4—C5—C6—C1	−0.3 (5)	C30—C25—C26—C27	−0.9 (4)
C2—C1—C6—C5	−2.1 (4)	C31—C25—C26—C27	−179.4 (2)
N1—C1—C6—C5	178.7 (3)	C25—C26—C27—C28	0.1 (4)
C1—N1—C7—N2	178.7 (2)	C26—C27—C28—C29	1.9 (5)
Zn1—N1—C7—N2	27.3 (4)	C26—C27—C28—N6	−177.2 (3)
C1—N1—C7—S1	2.3 (3)	O8—N6—C28—C27	13.7 (5)
Zn1—N1—C7—S1	−149.09 (14)	O7—N6—C28—C27	−168.6 (3)
C2—S1—C7—N1	−0.6 (2)	O8—N6—C28—C29	−165.4 (3)
C2—S1—C7—N2	−177.2 (2)	O7—N6—C28—C29	12.2 (5)
C15—N3—C9—C14	−178.7 (3)	C27—C28—C29—C30	−2.9 (5)
Zn1—N3—C9—C14	11.2 (4)	N6—C28—C29—C30	176.2 (3)
C15—N3—C9—C10	1.0 (3)	C28—C29—C30—C25	2.0 (5)
Zn1—N3—C9—C10	−169.03 (19)	C26—C25—C30—C29	−0.2 (4)
C14—C9—C10—C11	−1.0 (4)	C31—C25—C30—C29	178.3 (3)
N3—C9—C10—C11	179.3 (2)	Zn1—O6—C31—O5	−2.6 (4)
C14—C9—C10—S2	178.9 (2)	Zn1—O6—C31—C25	178.49 (16)
N3—C9—C10—S2	−0.9 (3)	C30—C25—C31—O5	−168.5 (3)
C15—S2—C10—C11	−179.8 (3)	C26—C25—C31—O5	10.0 (4)
C15—S2—C10—C9	0.4 (2)	C30—C25—C31—O6	10.5 (4)
C9—C10—C11—C12	1.0 (4)	C26—C25—C31—O6	−171.1 (2)

### Hydrogen-bond geometry (Å, °)

**Table:**

*D*—H···*A*	*D*—H	H···*A*	*D*···*A*	*D*—H···*A*
N2—H2A···O1	0.86	2.27	3.008 (3)	144.
N2—H2B···O5^i^	0.86	2.05	2.845 (3)	153.
N4—H4A···O6	0.86	2.20	2.971 (3)	149.
N4—H4B···O2^ii^	0.86	2.05	2.860 (3)	156.

Symmetry codes: (i) −*x*+1, −*y*, −*z*+1; (ii) −*x*, −*y*, −*z*+1.
